# Bifocal Spinal Cord Injury without Radiographic Abnormalities in a 5-Year Old Boy: A Case Report

**DOI:** 10.1155/2012/351319

**Published:** 2012-05-09

**Authors:** K. G. Snoek, M. Jacobsohn, A. B. van As

**Affiliations:** Department of Paediatric Surgery, Red Cross War Memorial Children's Hospital, University of Cape Town, Rondebosch, Cape Town 7701, South Africa

## Abstract

We present the extremely unusual case of a 5-year-old boy with a bifocal (cervical as well as lumbar) spinal cord injury without radiographic abnormalities (SCIWORAs). The MRI showed cord oedema at the level of C2 and T10. We propose that during the motor vehicle crash severe propulsion of the head with a flexed lumbar region resulted in a traction injury to the lower thoracic and lumbar spine and maximum flexion caused SCIWORA in C2.

## 1. Introduction

Spinal cord injury without radiographic abnormalities (SCIWORAs) is defined as objective signs and symptoms of myelopathy with normal plain radiographs and computed tomographic (CT) studies [1]. SCIWORA is a rare condition in children and typically involves the cervical cord [2]. Lumbar cord injuries are especially rare. It is estimated that from all spinal injuries 1 to 10% occur in childhood. The incidence of SCIWORA in children with spinal cord injury is over one-third of all cases. We present a 5-year-old boy with a bifocal (cervical as well as lumbar) SCIWORA. 

## 2. Case Report

**Figure 1:**
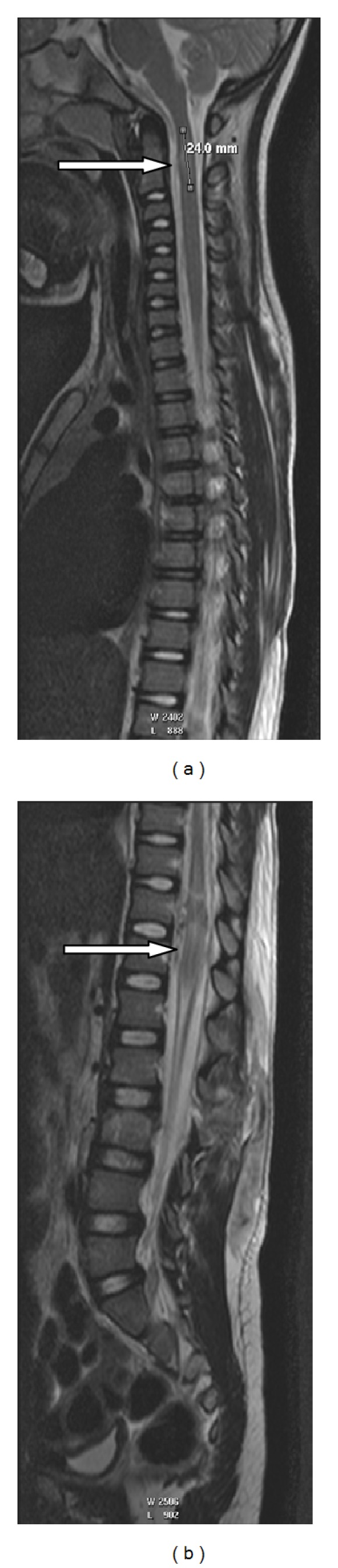
Lateral MRI images of the spinal cord at the cervical level (a) and the lumbar level (b). The spinal injuries are indicated with white arrows.

A 5-year-old boy presented to our hospital 2 days after being involved in a motor vehicle crash (MVC) with a velocity of 32 miles per hour. He was knocked down from the side while crossing the road as a pedestrian and was ejected about seven yards from the vehicle. On examination he had signs of torticollis to the right side, decreased reflexes and decreased power in his right arm (indicative of a brachial plexus injury), and decreased sensation below the clinical level of the second lumbar vertebra (L2). He also suffered decreased anal tone and lost bladder control. A Magnetic Resonance Image (MRI) demonstrated spinal cord oedema at the level of the second cervical vertebra (C2) (Figure 1(a)) as well as spinal cord injury with oedema from the level of the 10th thoracic vertebra till the first lumbar vertebra (T10-L1) (Figure 1(b)). There were also facet joint fractures with disrupted ligamentum flavum and interspinous ligaments at the level of the second and third vertebrae (L2-L3). Prior to the surgical procedure the cervical spine radiographs were normal.

**Figure 2:**
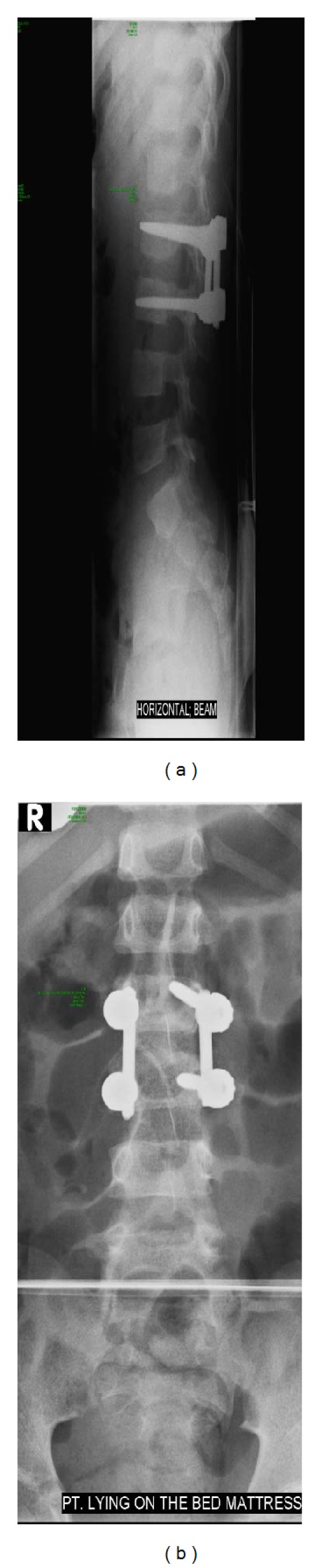
Lateral (a) and anteroposterior (b) radiographs of the lumbar spine after decompression of the spinal cord and fusion of second and third vertebral bodies (L2-L3).

After the MRI was performed, the patient underwent decompression of the spinal cord and fusion of the second and third vertebral bodies (L2-L3) (Figure 2). Afterwards he was admitted to the postoperative ICU for five days, where he developed right-sided paralysis of the diaphragm and subsequent pulmonary complications as well as a urinary tract infection. Computed Tomography (CT) scanning performed postoperatively demonstrated no further abnormalities. The outcome of this boy was good; he returned to normal life.

## 3. Discussion

While the paediatric vertebral column has the potential for significant stretch, this is unfortunately not matched by the spinal cord [3], leading to spinal cord injuries in SCIWORA. The cervical spine is at much higher risk of injury than the thoracic and lumbar spine in infants because of the large head and higher point of maximum flexion (C2-C3) [4]. The fulcrum for maximum flexion is at C2 to C3 in infants and young children [5]. The MVC caused maximum flexion in our patient with an SCIWORA at the level of C2 as a result. While upper cervical spine traction related injuries are relatively common, we propose that severe propulsion of the head with a flexed lumbar region resulted in a traction injury to the lower thoracic and lumbar spine [6]. Clinicians should have an index of suspicion for injury at a second level when one spinal injury is identified. A truly bifocal SCIWORA with segmental injuries (cervical and lumbar) of the spinal cord has not been reported in the literature to our knowledge. 

Since the MRI scan demonstrated edema at both foci, the prognosis of the injuries is relatively good according to the literature; in 75% of the patients with edema only on the MRI-scan the prognosis was scored as a mild grade, while 25% of patients returned to normal [5], which was the case in our patient one year after injury.
